# Single-time-point dosimetry using model selection and nonlinear mixed-effects modelling: a proof of concept

**DOI:** 10.1186/s40658-023-00530-1

**Published:** 2023-02-10

**Authors:** Deni Hardiansyah, Ade Riana, Ambros J. Beer, Gerhard Glatting

**Affiliations:** 1 Medical Physics and Biophysics, Physics Department, Faculty of Mathematics and Natural Sciences, Universitas Indonesia, Depok, Indonesia; 2 Research Collaboration Centre for Theranostic Radiopharmaceuticals, BRIN, Bandung, Indonesia; 3 Department of Nuclear Medicine, Ulm University, Ulm, Germany; 4 Medical Radiation Physics, Department of Nuclear Medicine, Ulm University, Albert-Einstein-Allee 23, 89081 Ulm, Germany

**Keywords:** Akaike weight, Model selection, NLME, PRRT

## Abstract

**Purpose:**

This project aims to develop and evaluate a method for accurately determining time-integrated activities (TIAs) in single-time-point (STP) dosimetry for molecular radiotherapy. It performs a model selection (MS) within the framework of the nonlinear mixed-effects (NLME) model (MS–NLME).

**Methods:**

Biokinetic data of [^111^In]In-DOTATATE activity in kidneys at *T*1 = (2.9 ± 0.6) h, *T*2 = (4.6 ± 0.4) h, *T*3 = (22.8 ± 1.6) h, *T*4 = (46.7 ± 1.7) h, and *T*5 = (70.9 ± 1.0) h post injection were obtained from eight patients using planar imaging. Eleven functions were derived from various parameterisations of mono-, bi-, and tri-exponential functions. The functions’ fixed and random effects parameters were fitted simultaneously (in the NLME framework) to the biokinetic data of all patients. The Akaike weights were used to select the fit function most supported by the data. The relative deviations (RD) and the root-mean-square error (RMSE) of the calculated TIAs for the STP dosimetry at *T*3 = (22.8 ± 1.6) h and *T*4 = (46.7 ± 1.7) h p.i. were determined for all functions passing the goodness-of-fit test.

**Results:**

The function $f_{4d} \left( t \right) = A_{1} /\left\{ {\left( {\frac{1 - \alpha }{{\lambda_{1} + \lambda_{{{\text{phys}}}} }}} \right) - \left( {\frac{\alpha }{{\lambda_{2} + \lambda_{{{\text{phys}}}} }}} \right) - \left( {\frac{1 - 2\alpha }{{\lambda_{bc} + \lambda_{{{\text{phys}}}} }}} \right)} \right\} \cdot e^{{ - \lambda_{{{\text{phys}}}} t}} \cdot \left\{ {\left( {1 - \alpha } \right) \cdot e^{{ - \lambda_{1} t}} - \alpha \cdot e^{{ - \lambda_{2} t}} - \left( {1 - 2\alpha } \right) \cdot e^{{ - \lambda_{bc} t}} } \right\}$ with four adjustable parameters and $\lambda_{bc} = \frac{{{\text{ln}}\left( 2 \right)}}{{1\;{\text{ min}}}}$ was selected as the function most supported by the data with an Akaike weight of (45 ± 6) %. RD and RMSE values show that the MS–NLME method performs better than functions with three or five adjustable parameters. The RMSEs of TIA_NLME–PBMS_ and TIA_3-parameters_ were 7.8% and 10.9% (for STP at T3), and 4.9% and 10.7% (for STP at T4), respectively.

**Conclusion:**

An MS–NLME method was developed to determine the best fit function for calculating TIAs in STP dosimetry for a given radiopharmaceutical, organ, and patient population. The proof of concept was demonstrated for biokinetic ^111^In-DOTATATE data, showing that four-parameter functions perform better than three- and five-parameter functions.

**Supplementary Information:**

The online version contains supplementary material available at 10.1186/s40658-023-00530-1.

## Introduction

Individual dosimetry in molecular radiotherapy (MRT) is often not performed in the clinic, as it requires sequential imaging to determine the time-integrated activity (TIA) [1–4]. These repeated measurements need several patient visits resulting in an additional burden for the patients and costs for the clinics. Therefore, developing methods to simplify individual dosimetry by reducing the total number of measurements is highly desirable.

Several studies have investigated the feasibility of using a low number of measurements for the determination of TIAs in radioimmunotherapy [5], radioiodine therapy [6, 7], peptide-receptor radionuclide therapy (PRRT) [3, 6, 8–15], and [^177^Lu]Lu-PSMA therapy [16, 17]. Recently, Devasia et al. implemented a nonlinear mixed-effect (NLME) model to determine the TIAs of [^177^Lu]Lu-DOTATATE in kidneys during PRRT using a bi-exponential function and single-time-point (STP) imaging with SPECT/CT [8]. As a result, the NLME model was able to lower the number of TIAs with a bias > 10% (32 of 500 simulations, 6%) by a factor of about three compared to the number of TIAs with a bias > 10% obtained from dose mapping methods introduced by Hänscheid et al. [9] (102 of 500 simulations, 20%), and the curve-fitting methods introduced by Madsen et al. [12] (85 of 500 simulations, 17%). Furthermore, we showed that individual dosimetry based on STP data, NLME, and a PBPK model could lead to relatively accurate determination of TIAs in various organs [15].

Although STP imaging and NLME modelling has shown promising results, using a bi-exponential function [8] might not be optimal for all radiopharmaceuticals, organs, or patient populations. For example, it has been demonstrated that model selection is a crucial step in estimating TIAs in MRT [18], as estimating TIAs highly depends on the chosen fit function [18, 19]. In this study, we developed and performed a model selection with NLME (MS–NLME) modelling to determine the fit function best supported by our data set as a proof of concept. Then, we compared the performance of the best fit function from NLME-PBMS to the performance of the bi-exponential function [8, 19, 20] in determining TIAs in STP dosimetry.

## Materials and methods

### Biokinetic data

In brief, biokinetic data of [^111^In]In-DOTATATE in kidneys from eight patients with either meningioma (*n* = 4) or neuroendocrine tumours (*n* = 4) were used in this study [15, 21]. An activity of (140 ± 14) MBq of [^111^In]In-DOTATATE was administered intravenously to the patients as a (51 ± 8) min infusion. Planar whole-body scintigraphies using a double-head gamma camera (ECAM, Siemens, Erlangen, Germany) were performed at *T*1 = (2.9 ± 0.6) h, *T*2 = (4.6 ± 0.4) h, *T*3 = (22.8 ± 1.6) h, *T*4 = (46.7 ± 1.7) and *T*5 = (70.9 ± 1.0) h p.i. [21] using a medium energy collimator with energy windows *A*1 = 171 keV (width 15%), *A*2 = 245 keV (15%), *B*1 = 142 keV (18%), and *B*2 = 205 keV (18%). Background correction and self-attenuation were included in the measurement of organ activity as a function of time according to the MIRD pamphlet number 16 [22]. The percentage of the administered activity in kidneys was used in this study [21]. Biokinetic data of [^111^In]In-DOTATATE were used as a surrogate for predicting the kinetics of [^90^Y]Y-DOTATATE used for peptide-radionuclide therapy, as suggested in the literature [21, 23].

### Sums of exponential functions

In this study, the following sums of exponential (SOEs) functions with 3, 4, and 5 parameters and different parameterisations were used to fit the biokinetics of [^111^In]In-DOTATATE in kidneys (Eqs. 1–11). The different parameterisations were investigated to demonstrate that the NLME modelling yields different results for different parameterisations.

1 $$f_{3a} \left( t \right) = A_{1} e^{{ - \left( {\lambda_{1} + \lambda_{{{\text{phys}}}} } \right)t}} - A_{1} e^{{ - \left( {\lambda_{2} + \lambda_{{{\text{phys}}}} } \right)t}}$$

2 $$f_{3b} \left( t \right) = \frac{{\left( {\left( {\lambda_{1} + \lambda_{{{\text{phys}}}} } \right) \times \left( {\lambda_{2} + \lambda_{{{\text{phys}}}} } \right)} \right)}}{{A_{1} \left( {\left( {\lambda_{2} + \lambda_{{{\text{phys}}}} } \right) - \left( {\lambda_{1} + \lambda_{{{\text{phys}}}} } \right)} \right)}}\left[ {e^{{ - \left( {\lambda_{1} + \lambda_{{{\text{phys}}}} } \right)t}} - e^{{ - \left( {\lambda_{2} + \lambda_{{{\text{phys}}}} } \right)t}} } \right]$$

3 $$f_{3c} \left( t \right) = A_{1} \frac{{\left( {\left( {\lambda_{1} + \lambda_{{{\text{phys}}}} } \right) \times \left( {\lambda_{2} + \lambda_{{{\text{phys}}}} } \right)} \right)}}{{\left( {\left( {\lambda_{2} + \lambda_{{{\text{phys}}}} } \right) - \left( {\lambda_{1} + \lambda_{{{\text{phys}}}} } \right)} \right)}}\left[ {e^{{ - \left( {\lambda_{1} + \lambda_{{{\text{phys}}}} } \right)t}} - e^{{ - \left( {\lambda_{2} + \lambda_{{{\text{phys}}}} } \right)t}} } \right]$$

4 $$f_{3d} \left( t \right) = \frac{{\left( {\lambda_{1} \times \lambda_{2} } \right)}}{{A_{1} \left( {\lambda_{2} - \lambda_{1} } \right)}}\left[ {e^{{ - \left( {\lambda_{1} } \right)t}} - e^{{ - \left( {\lambda_{2} } \right)t}} } \right]$$

5 $$f_{4a} \left( t \right) = A_{1} e^{{ - \left( {\lambda_{1} + \lambda_{{{\text{phys}}}} } \right)t}} - A_{2} e^{{ - \left( {\lambda_{2} + \lambda_{{{\text{phys}}}} } \right)t}} - \left( {A_{1} - A_{2} } \right) - e^{{ - \left( {\lambda_{bc} + \lambda_{{{\text{phys}}}} } \right)t}}$$

6 $$f_{4b} \left( t \right) = A_{1} \alpha e^{{ - \left( {\lambda_{1} + \lambda_{{{\text{phys}}}} } \right)t}} - A_{1} \left( {1 - \alpha } \right)e^{{ - \left( {\lambda_{2} + \lambda_{{{\text{phys}}}} } \right)t}} - A_{1} \left( {2\alpha - 1} \right)e^{{ - \left( {\lambda_{bc} + \lambda_{{{\text{phys}}}} } \right)t}}$$

7 $$f_{4c} \left( t \right) = A_{1} \left( {1 - \alpha } \right)e^{{ - \left( {\lambda_{1} + \lambda_{{{\text{phys}}}} } \right)t}} - A_{1} \alpha e^{{ - \left( {\lambda_{2} + \lambda_{{{\text{phys}}}} } \right)t}} - A_{1} \left( {1 - 2\alpha } \right)e^{{ - \left( {\lambda_{bc} + \lambda_{{{\text{phys}}}} } \right)t}}$$

8 $$f_{4d} \left( t \right) = \frac{{A_{1} }}{{\left\{ {\left( {\frac{1 - \alpha }{{\lambda_{1} + \lambda_{{{\text{phys}}}} }}} \right) - \left( {\frac{\alpha }{{\lambda_{2} + \lambda_{{{\text{phys}}}} }}} \right) - \left( {\frac{1 - 2\alpha }{{\lambda_{bc} + \lambda_{{{\text{phys}}}} }}} \right)} \right\}}}e^{{ - \lambda_{{{\text{phys}}}} t}} \left\{ {\left( {1 - \alpha } \right)e^{{ - \lambda_{1} t}} - \alpha e^{{ - \lambda_{2} t}} - \left( {1 - 2\alpha } \right)e^{{ - \lambda_{bc} t}} } \right\}$$

9 $$f_{4e} \left( t \right) = \frac{{A_{1} }}{{\left\{ {\left( {\frac{\alpha }{{\lambda_{1} + \lambda_{{{\text{phys}}}} }}} \right) - \left( {\frac{1 - \alpha }{{\lambda_{2} + \lambda_{{{\text{phys}}}} }}} \right) - \left( {\frac{2\alpha - 1}{{\lambda_{bc} + \lambda_{{{\text{phys}}}} }}} \right)} \right\}}}e^{{ - \lambda_{{{\text{phys}}}} t}} \left\{ {\alpha e^{{ - \lambda_{1} t}} - \left( {1 - \alpha } \right)e^{{ - \lambda_{2} t}} - \left( {2\alpha - 1} \right)e^{{ - \lambda_{bc} t}} } \right\}$$

10 $$f_{5a} \left( t \right) = A_{1} e^{{ - \left( {\lambda_{1} + \lambda_{{{\text{phys}}}} } \right)t}} - A_{2} e^{{ - \left( {\lambda_{2} + \lambda_{{{\text{phys}}}} } \right)t}} - \left( {A_{1} - A_{2} } \right)e^{{ - \left( {\lambda_{3} + \lambda_{{{\text{phys}}}} } \right)t}}$$

11 $$f_{5b} \left( t \right) = A_{1} e^{{ - \left( {\lambda_{1} + \lambda_{{{\text{phys}}}} } \right)t}} + A_{2} e^{{ - \left( {\lambda_{{{\text{phys}}}} } \right)t}} - A_{3} e^{{ - \left( {\lambda_{2} + \lambda_{{{\text{phys}}}} } \right)t}} - \left( {A_{1} + A_{2} - A_{3} } \right)e^{{ - \left( {\lambda_{bc} + \lambda_{{{\text{phys}}}} } \right)t}}$$

where $f_{i}$ is a fit function, $i$ is the total number of the estimated parameters, $A_{j} \left( {j = 1,2,3} \right)$ are the prefactors of the fit function with values $\ge 0$, $\lambda_{{{\text{phys}}}}$ is the physical decay constant of ^111^In ($\lambda_{{{\text{phys}}}} = \ln \left( 2 \right)/T_{1/2} = 1.72 \times 10^{ - 4} \min^{ - 1}$ [21]), $\lambda_{bc}$ is the rate of blood circulation of 1 min $\left( {\lambda_{bc} = \frac{\ln \left( 2 \right)}{{1 \min }}} \right)$, $\lambda_{j}$ are the biological decay constants of the radiopharmaceutical with values $\ge 0$, and the $\alpha$ values are the fractional contributions of the corresponding exponentials with values between 0 and 1. As described in Burnham et al. [24], existing prior knowledge should be taken into account when selecting the functions to be used for model selection. Therefore, on the one hand only sums of exponential functions were considered [19, 25] and on the other hand the constraint $f_{i} \left( {t = 0} \right) = 0$ was implemented. In addition, for functions with 4 and 5 parameters, a rapid increase in activity in the kidneys with a half-life of 1 min was added, which is caused by the blood circulation time in humans. SOE functions with less than three parameters did not pass the goodness-of-fit test and were not included in the analysis.

### Nonlinear mixed-effects model

Parameters in the NLME model consist of the fixed and random effects (Eqs. 12–13) as reported in the literature [8, 15, 26]. Fixed effects describe the mean values of the estimated parameters in the population, while random effects describe the inter-patient variability of the estimated parameters between subjects in the population [27].

12 $$P_{j} = {\text{TVP}}_{j} \times \exp \left( {{\text{ETA}}_{j} } \right)$$

13 $${\text{ETA}}_{j} = N\left( {0,\sigma_{j}^{2} } \right)$$

where $P_{j}$ is the estimated parameter $j$ in an SOE function, ${\text{TVP}}_{j}$ is the fixed effect of the estimated parameter $j$, and ${\text{ETA}}_{j}$ the random effect. ${\text{ETA}}_{j}$ is a random number following a Gaussian distribution with mean zero and variance $\sigma_{i}^{2}$. Parameters of the exponential functions in Eqs. (1–11) were modelled as the combination of a fixed effect and an inter-patient variability (random effect) plus the intra-patient variability.

### Study workflow

The parameters of the SOE functions (Eqs. 1–11) were fitted to the biokinetic data of [^111^In]In-DOTATATE in kidneys (“Biokinetic data” Section) using the NLME method. All NLME model fittings and simulations were performed in MATLAB software vR2020a. As suggested in the literature, an exponential error model with log transformation was used [15]. The MS–NLME method is performed using the Akaike weight. The SOE function with the highest Akaike weight was selected as the fit function most supported by the data. The Akaike weights indicate the probability that the model is the best among the analysed models [24, 28]. The Akaike weights [19, 24, 28] of the SOE functions were calculated as follows:

14 $${\text{AICc}} = - 2\ln \left( P \right) + 2K + \frac{{2K\left( {K + 1} \right)}}{N - K - 1}$$

15 $$\Delta_{j} = {\text{AICc}}_{j} - {\text{AICc}}_{\min }$$

16 $$w_{{{\text{AICc}}_{j} }} = e^{{\frac{{ - \Delta_{j} }}{2}}} /\mathop \sum \limits_{i = 1}^{F} e^{{\frac{{ - \Delta_{i} }}{2}}}$$

where ${\text{AICc}}$ is the corrected Akaike Information Criterion value, $P$ is the obtained minimum objective function, ${\text{AICc}}_{\min }$ is the lowest ${\text{AICc}}$ value of the SOE functions, $\Delta_{j}$ is the difference of the ${\text{AICc}}_{j}$ of SOE function $j$ and ${\text{AICc}}_{\min }$, $F$ is the number of SOE functions in the model set, and $w_{{{\text{AICc}}_{j} }}$ is the Akaike weight of function $j$. The stability of the best SOE function obtained from the MS–NLME method was tested using the Jackknife method [28, 29]. In this method, the leave-one-out method was applied eight times with only seven patients included for the calculation of the Akaike weights.

The performance in determining the TIAs for STP dosimetry of the best SOE function obtained from the MS–NLME method was compared to the performance of the often used bi-exponential function $f_{3d}$ [8, 19, 20]. The parameters of the bi-exponential function ($f_{3d}$ Eq. (4)) were fitted to a patient with only STP biokinetic data by simultaneously fitting within the NLME model framework this new patient’s limited measurement with all data points of all other patients. Biokinetic data at time points *T*3 = (22.8 ± 1.6) and *T*4 = (46.7 ± 1.7) p.i. were used for the STP fitting as suggested in the literature [15].

TIAs from the STP fitting using the bi-exponential function ($f_{3d}$) were calculated by integrating the individual simulated time-activity curves from *t* = 0–100,000 min (TIA_STP_f3d_). The STP NLME model fittings were repeated using the best model obtained from MS–NLME method, followed by calculating the corresponding TIAs (TIA_STP_MS–NLME_).

Relative deviations (RDs) and root-mean-square errors (RMSEs) were used to analyse the accuracy of the calculated TIA_STP_f3d_ and TIA_STP_NLME–PBMS_ with the TIAs obtained from the all-time-point fittings using the best model obtained from MS–NLME (TIA_ATP_MS–NLME_) as the reference. The relative deviation RDs and the RMSEs were calculated according to

17 $${\text{RD}}_{k,m} = \frac{{{\text{TIA}}_{k,m} - {\text{TIA}}_{{{\text{ATPMS}} - {\text{NLME}},m}} }}{{{\text{TIA}}_{{{\text{ATPMS}} - {\text{NLME}},m}} }},$$

18 $${\text{RMSE}}_{k} = \sqrt {\left( {{\text{SDRD}}_{k,m} } \right)^{2} + \left( {{\text{MeanRD}}_{k,m} } \right)^{2} } ,$$

where ${\text{RD}}_{k,m}$ is the relative deviation of STP method $k$ of patient $m$_,_ ${\text{RMSE}}_{k}$ is the root-mean square over all patients of ${\text{RD}}_{k,m}$, ${\text{SDRD}}_{k,m}$ is the standard deviation of ${\text{RD}}_{k,m}$, ${\text{MeanRD}}_{k,m}$ is the mean of ${\text{RD}}_{k,m}$, and $k$ determines the function used for the NLME modelling.

## Results

Based on the MS–NLME approach, function $f_{4d}$ was selected as the function most supported by the data with an Akaike weight of 47.26% (Table 1). Function $f_{4e}$ was ranked as the second-best function with an Akaike weight of 44.25%. All bi-exponential functions with three parameters had Akaike weights ≤ 0.10%. The Jackknife method was applied to the subset of functions with Akaike weights > 1%, i.e. $f_{4b}$, $f_{4d}$, and $f_{4e}$ (Table 1). Based on the Jackknife method, the selection of $f_{4d}$ was stable with median Akaike weight of 43% and range 38–56% (Table 1). Functions $f_{4c}$, $f_{5a}$, and $f_{5b}$ did not pass the goodness-of-fit test with maximum CV of the estimated fixed effect > 50%.

**Table 1:** The goodness of fits and Akaike weights for the investigated functions in the NLME model

Equation number	Function name	*K*	Akaike weight (%)	Jackknife Akaike weights (% mean (SD); % median [min, max])^a^	RD (% median [min, max])^b^	RMSE of the RD (%)^b^
1	$f_{3a}$	7	0.08	–	− 3.94 [− 4.39, − 2.05]	3.66
2	$f_{3b}$	7	0.09	–	− 3.79 [− 5.99, − 2.19]	4.06
3	$f_{3c}$	7	0.09	–	− 3.89 [− 4.61, − 2.16]	3.69
4	$f_{3d}$	7	0.11	–	− 3.64 [− 6.17, − 2.28]	4.04
5	$f_{4a}$	9	0.77	–	− 0.78 [− 1.72, 0.56]	0.97
6	$f_{4b}$	9	7.35	10 (7); 11 [0,21]	− 0.69 [− 1.91, 0.44]	1.09
7	$f_{4c}$ ^c^	9	–	–	–	–
8	$f_{4d}$	9	47.26	45 (6); 43 [38,56]	–	–
9	$f_{4e}$	9	44.25	45 (7); 43 [37,57]	− 0.49[− 0.64,− 0.04]	0.49
10	$f_{5a}$ ^c^	11	–	–	–	–
11	$f_{5b}$ ^c^	11	–	–	–	–

The total number of biokinetic data *N* used in this analysis is 40; the number of parameters of the NLME model for the corresponding SOE function is given in column *K*

^a^The Jackknife analysis was based on functions with Akaike weights > 1%, i.e. $f_{4b}$, $f_{4d}$, and $f_{4e}$

^b^Function $f_{4d}$ was used as the reference for the calculation of the RDs and RMSEs of the all-time-point TIAs

^c^Functions $f_{4c}$, $f_{5a}$, and $f_{5b}$ did not pass the goodness-of-fit test because CV > 50%; therefore, Akaike weights, RDs, and RMSEs were not calculated for these functions

**Fig. 1:**
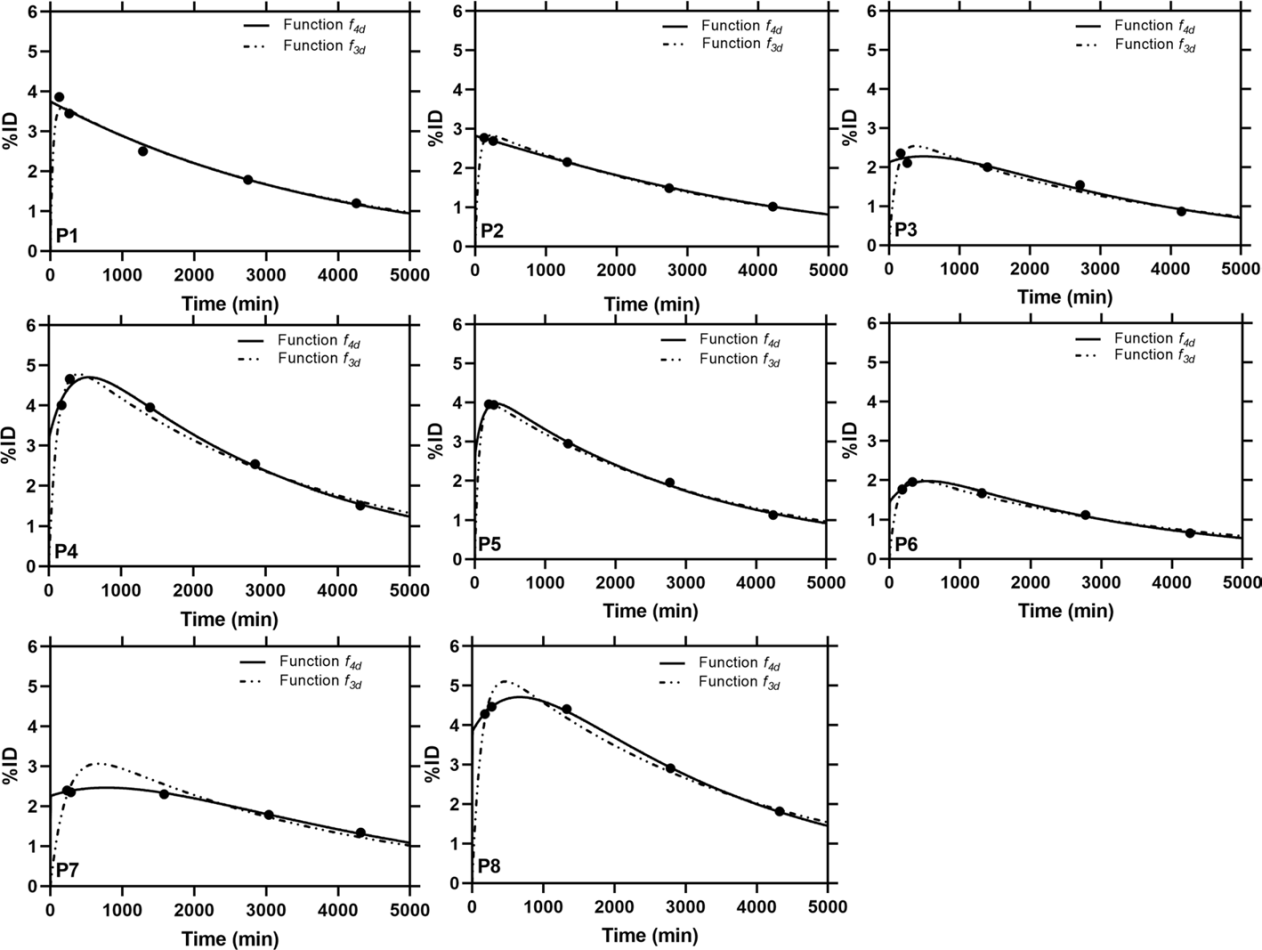
Time-activity data and ATP fit curves obtained using function $f_{4d}$, which is chosen as most supported by the data using the presented MS–NLME method. Function $f_{3d}$ is shown for comparison, as it is the function with the highest Akaike weight from the group of functions with three and five parameters

**Fig. 2:**
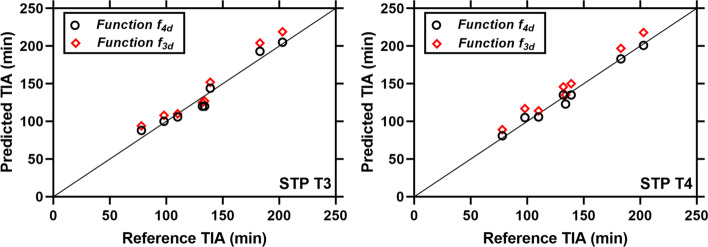
Comparison of the predicted TIAs calculated from STP measurements at *T*3 or *T*4 with functions $f_{4d}$ and $f_{3d}$. Reference TIAs were calculated using the all-time-point data and function $f_{4d} .$

Figure 1 compares the time-activity curves of the best function obtained from the MS–NLME ($f_{4d}$) with the function $f_{3d}$. Visual inspection of the fitted graphs in Fig. 1 shows that function $f_{4d}$ has a better performance than function $f_{3d}$. All fitted parameters using the MS–NLME function ($f_{4d}$) showed a precise value with a coefficient of variation < 50% (Table 2). Predicted TIAs of STP dosimetry using function $f_{4d}$ showed better results in most of the patients compared to the predicted TIAs of STP dosimetry using function $f_{3d}$ (Fig. 2).

**Table 2:** Parameters estimated from ATP fitting using the best model obtained from the best function derived using MS–NLME method, i.e. $f_{4d}$

Model parameters	Fixed effect (% coefficient of variation)	Random effect (variance, inter-patient variability)
$A$	129.36 (10.5) min	0.08
$\alpha$	0.31 (7.6)	0.01
$\lambda_{1}$^a^	1.5 × 10^–4^ (9.8)/min	2.9 × 10^−4^
$\lambda_{2}$^b^	8.8 × 10^–4^ (41.4)/min	1.1
Intra-patient variability	4.4 × 10^–2^ (21)	–

^a^Biological decay $\lambda_{1}$ corresponds to biological half-life $T_{1/2}$ = (77.0 ± 0.8) h

^b^Biological increase $\lambda_{2}$ corresponds to biological half-life $T_{1/2}$ = (13.1 ± 0.1) h

**Fig. 3:**
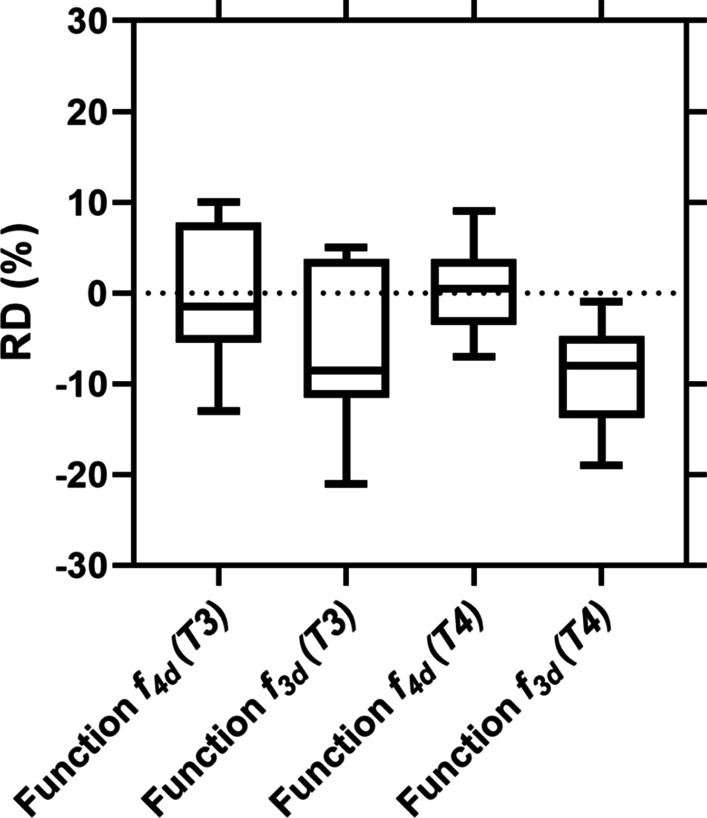
RDs of TIAs obtained from the function $f_{4d}$ and $f_{3d}$ for STP dosimetry at *T*3 and T4. TIAs from all-time-point fittings calculated using function $f_{4d}$ were used as the reference values

Figure 3 and Additional file 1: Table S1 show the RD and RMSE of the TIAs of the MS–NLME ($f_{4d}$) function and function $f_{3d}$ to the reference TIAs calculated from all-time-point fitting (“Study Workflow” Section). The MS–NLME ($f_{4d}$) function has a better performance than the function $f_{3d}$ by a factor of two based on the RMSEs values (Additional file 1: Table S1) for STP dosimetry at T4. Figure 3 shows the %RD of TIAs from STP dosimetry using functions $f_{4d}$ and $f_{3d}$. As a result, the RMSE value of function $f_{4d}$ (4.9%) is lower by a factor of 2 compared to when using function $f_{3d}$ (10.7%) (Fig. 3 and Additional file 1: Table S1).

## Discussion

The NLME model is a promising population-based method for calculating TIAs in MRT [8, 15]. Implementation of the NLME model using a bi-exponential function ($f_{3d}$) in STP dosimetry showed a better result [8] than the STP methods introduced by Hänscheid et al. [9] and Madsen et al. [12]. However, using a bi-exponential function in STP dosimetry [8] might not be optimal for all radiopharmaceuticals, organs, or patient data sets: It will depend on the biokinetic data set, e.g. the number and schedule of the time-activity data, the radiopharmaceutical, and the organ. Different groups have used various exponential functions in their STP dosimetry analyses [8, 9, 12]. The lack of standardised methods for finding functions for calculating TIAs might lead to reproducibility issues [18]. To find the function that best describes the biokinetic data model selection is needed for reproducible and optimal estimation of TIAs in STP. In this study, we developed the MS–NLME method and investigated the effect of choosing the best function on the accuracy of TIAs in STP dosimetry in our data set. As a result, we showed with our data set that this method offers, on the one hand, a more reproducible approach than choosing a function based on a simple rule of thumb and, on the other hand, also improves the achieved TIA accuracy.

Determination of the set of functions is a critical step in model selection. To get a good model selection output, the functions in the set of model functions should include prior knowledge [24], for example, about the physiology of the considered organ. Therefore, characteristics of the functions used in our study based on the prior knowledge of the biokinetic data in kidneys were (1) the value of $f_{i} \left( {t = 0} \right) = 0$, and (2) a fast uptake of $T_{1/2}$ = 1 min was used for functions $f_{4a}$, $f_{4b}$, $f_{4c}$, $f_{4d}$, and $f_{4e}$. This fast uptake was fixed, as it could not be fitted because the first measured time point was *T*1 = (2.9 ± 0.6) h.

Exponential functions with one and two parameters are not presented here as they either did not pass the goodness-of-fit criteria (Table 1 in [19]) or had a negligible Akaike weight. In general, functions with four parameters have relatively higher Akaike weight compared to that of functions with three and five parameters (Table 1). Function $f_{4d}$ was the best function of the investigated functions with four parameters. Based on the Jackknife analysis results, functions $f_{4d}$ and $f_{4e}$ have almost an equal performance with Akaike weights of median [min, max] of 43% [38%, 56%] and 43% [37%, 57%], respectively. This is a consequence of the high similarity of both functions, which are just two different parameterisations of the same underlying function. Therefore, the NLME model fits also look indistinguishable (Additional file 1: Figure S2), although there is a difference in the TIAs from the all-time-point fittings from both functions; consequently, it is not shown in Fig. 1. This result shows the importance of having different parameterisations of the same function in the model set when applying the NLME model. Also, differences between different functions are larger compared to differences between the various parameterisations of the same function (Table 1).

Functions with five parameters had a high coefficient of variation of the fitted fixed effect (> 50%) and were thus not included in the calculation of Akaike weights (Table 1). These could be attributed to the limited number of data used in this study which were not enough to estimate two biological decay rates ($f_{5a}$) or two biological uptake rates ($f_{5b}$).

Function $f_{4d}$ was selected as the best function for our data set based on the Akaike weight (Table 1). Function $f_{3d}$ was used as the function of interest to analyse the performance of function $f_{4d}$ because [1] function $f_{3d}$ has the highest Akaike value of all investigated functions with three and five parameters, and [2] function $f_{3d}$ was used for the STP dosimetry with NLME modelling in the literature [8]. Visual inspection of the individual all-time-point fitted graphs in Fig. 1 (especially P7 and P8) shows that function $f_{4d}$ performs better than function $f_{3d}$. Furthermore, STP dosimetry using function $f_{4d}$ has superior performance than STP dosimetry using function $f_{3d}$ in predicting the TIAs (Additional file 1: Table S1, Figs. 2 and 3). Presumably, when a better fit function is used, the accuracy and precision of STP dosimetry also become higher. Model averaging was not used in this study as it would not change the major finding that 3- and 5-parameter functions are less good than 4-parameter functions.

More sophisticated models for describing the kinetics of radiopharmaceuticals, e.g. physiologically based pharmacokinetic models [3, 30, 31], can also be used to investigate the effect of the model selection to the STP dosimetry. This would allow for incorporating more knowledge of pharmacokinetic and physiological processes. However, most practitioners of dosimetry use mono-, bi-, and tri-exponentials for fitting the time-activity data [8, 19, 20, 25, 32]. Therefore, in this study, we decided to show how to apply the population-based model selection method for sums of exponential functions.

A limitation of our proof-of-concept study is that the total number of patients included in this study is relatively low. However, even for this small number of patients, the Akaike weight uncertainty using the Jackknife method demonstrates a clear priority for functions with 4 parameters. This translates into a benefit also reflected in the lower value of the RDs for the corresponding functions (Fig. 3). Nevertheless, further studies are needed with larger sets of patient biokinetic data for various radiopharmaceuticals and organs.

Biokinetic data of [^111^In]In-DOTATATE were used for the proof of concept of STP approach with NLME modelling plus a model selection for the fit function. Biokinetic data of [^111^In]In-DOTATATE can be used as a surrogate for predicting the kinetics of [^90^Y]Y-DOTATATE [20, 22].

In the clinical setting, the presented STP method would be essential in the sequence of steps as follows:

1. Collect biokinetic data of a patient population either from pretherapeutic or therapeutic measurements,
2. Derive the fit function most supported by the data according to the here presented MS–NLME method.
3. Perform STP dosimetry for “new” patients using the derived best function and the NLME model fitting with the inter- and intra-individual variabilities determined in item 2.

## Conclusions

To determine the best fitting function for calculating TIAs in STP dosimetry for a given radiopharmaceutical, organ and patient population, we proposed a population-based model selection method (MS–NLME). The application of this method was demonstrated for the biokinetics of [^111^In]In-DOTATATE as proof of concept: Since STP dosimetry depends on the fit function used, determining the best fit function is essential for an optimal STP dosimetry method. In general, NLME modelling, a standard procedure in pharmacokinetic science, is a promising approach to individualise MRT dosimetry by STP measurements.

## Supplementary Information

**Additional file 1.** Time-activity curves, relative deviations, and root-mean-square error of sum of exponentials functions.

## Data Availability

The used data are available from the corresponding author upon reasonable request.

## Abbreviations

ATP: All-time-point
MRT: Molecular radiotherapy
MS–NLME: Model selection with NLME
NLME: Nonlinear mixed-effects
PRRT: Peptide-receptor radionuclide therapy
RD: Relative deviations
RMSE: Root-mean-square error
SOE: Sum of exponentials
STP: Single-time-point
TIA: Time-integrated activity
