# Supplementary material for: Single-time-point dosimetry using model selection and nonlinear mixed-effects modelling: a proof of concept
Source: EJNMMI Phys. 2023 Feb 10;10:12. doi: 10.1186/s40658-023-00530-1 (PMC9911583; doi:10.1186/s40658-023-00530-1)
Supplement: Supplementary file 1 — Additional file 1. Time-activity curves, relative deviations, and root-mean-square error of sum of exponentials functions. [file 40658_2023_530_MOESM1_ESM.pdf]

Supplemental File

Single-Time-Point Dosimetry Using Model Selection and Non-Linear Mixed-Effects Modelling: A Proof of Concept

Deni Hardiansyah[1,2], Ade Riana[1], Ambros J. Beer[3], Gerhard Glatting[3,4]

[1]Medical Physics and Biophysics, Physics Department, Faculty of Mathematics and Natural Sciences, Universitas Indonesia, Depok, Indonesia

[2]Research Collaboration Center for Theranostic Radiopharmaceuticals, Bandung, Indonesia

[3]Department of Nuclear Medicine, Ulm University, Ulm, Germany

[4]Medical Radiation Physics, Department of Nuclear Medicine, Ulm University, Ulm, Germany

Fig. S1

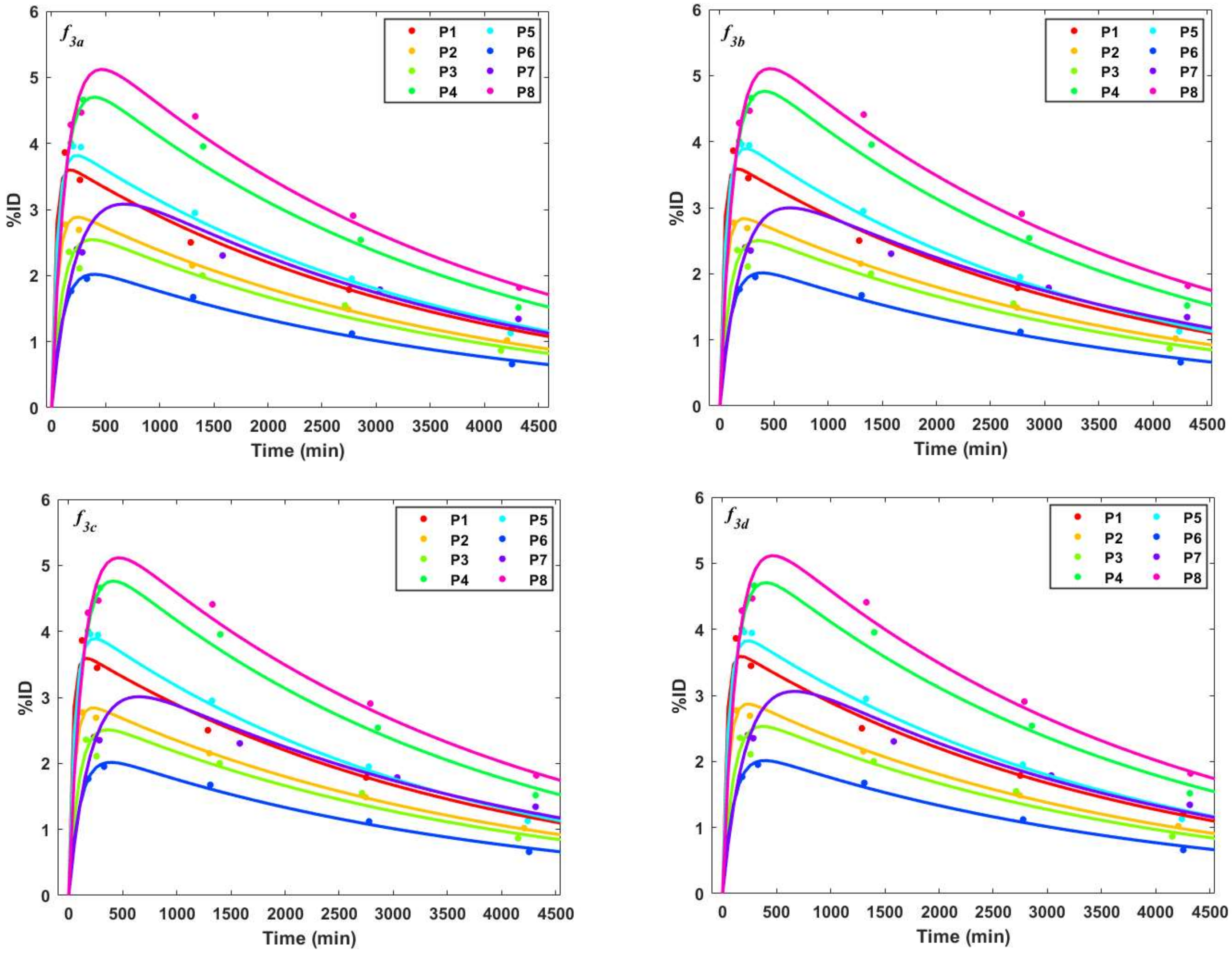

Fig. S1. Time-activity data and all-time-points (ATP) fit curves obtained using functions f_{3a}, f_{3b}, f_{3c}, and f_{3d}.

Fig. S2

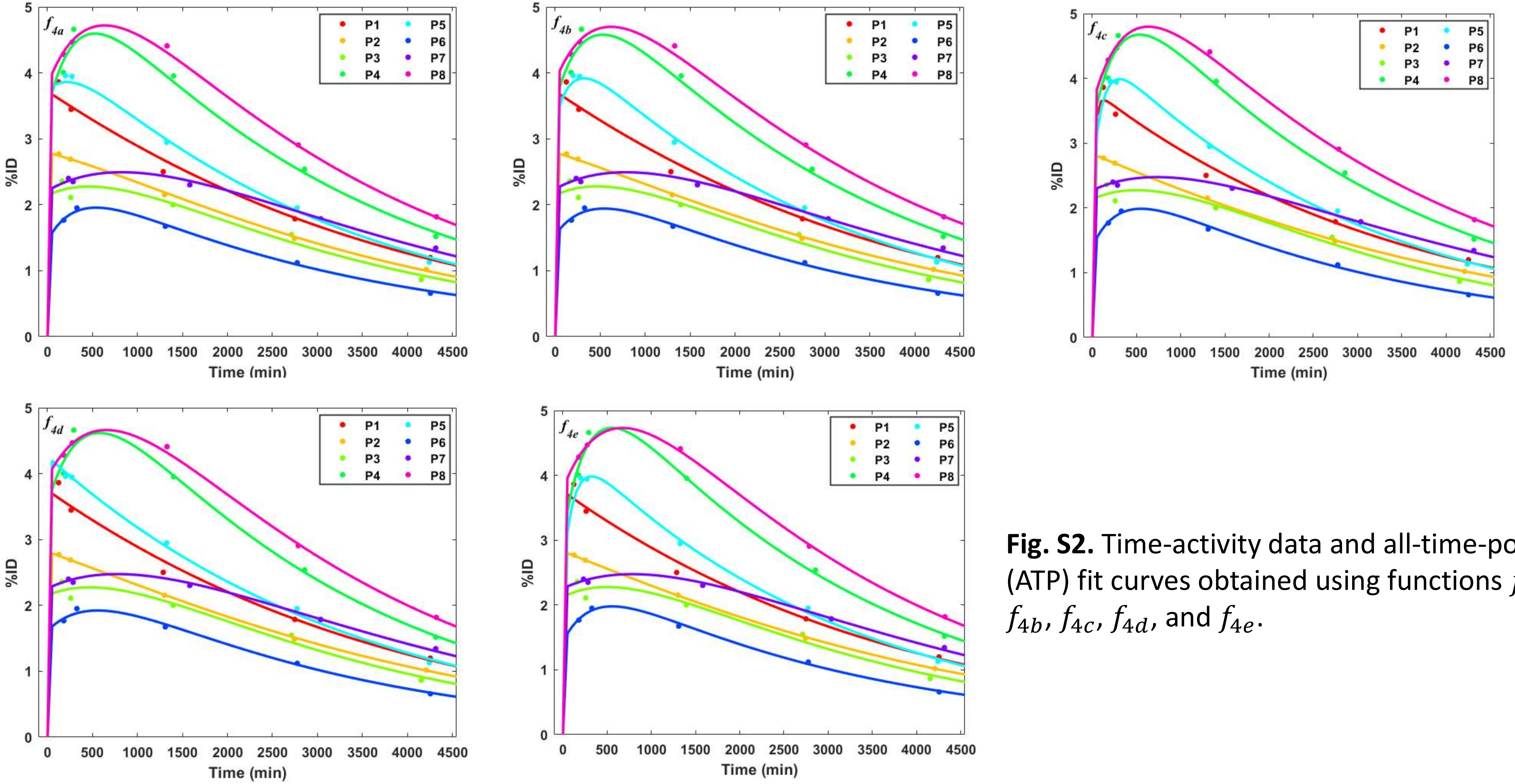

Fig. S2. Time-activity data and all-time-points (ATP) fit curves obtained using functions f_{4a}, f_{4b}, f_{4c}, f_{4d}, and f_{4e}.

Fig. S3

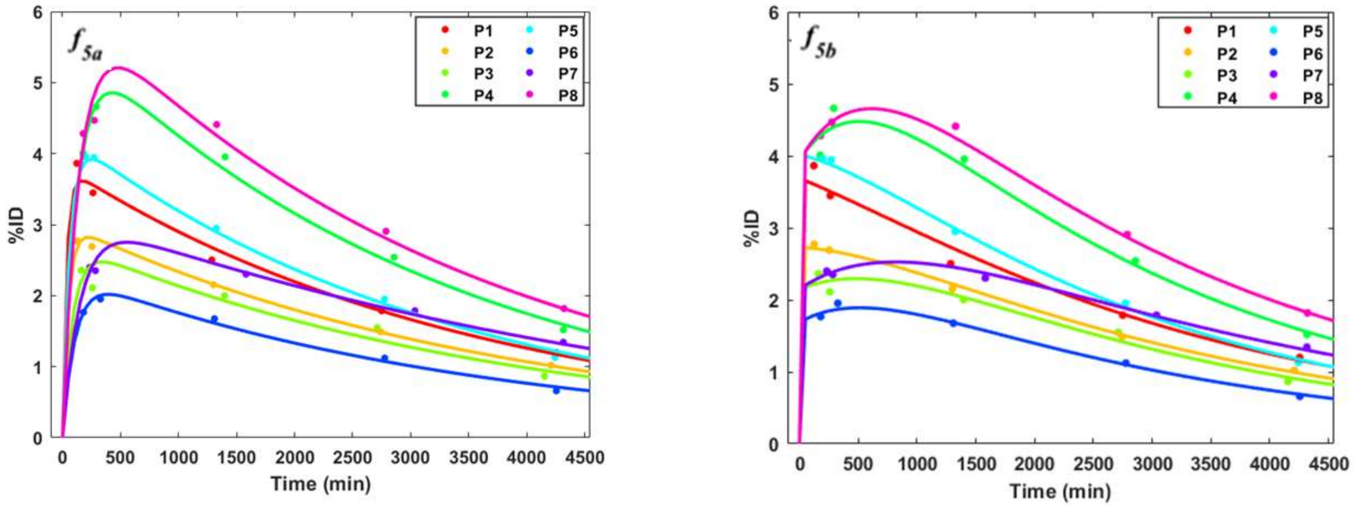

Fig. S3. Time-activity data and all-time-points (ATP) fit curves obtained using functions f_{5a}, and f_{5b}.

Tab. S1. RDs and RMSEs of TIAs obtained using a single time point fitting, and the best function of MS-NLME (f_{4d}) and the 3-parameter function f_{3d} with the second-highest Akaike weight (Table 1) of the 3-parameter functions (also used in ref. [8]). The RMSE of the STP method with the MS-NLME best function has a better performance than the RMSE of the STP method with function f_{3d} by a factor of two, which shows the superiority of the MS-NLME approach over the non-model-selecting NLME approach.

STP Methods	Timepoint (h)	RD (%)		RMSE of the %RD (%)
		mean (SD)	median [min, max]	
Function f_{4d}	T3 (22.8±1.6)	0 (8)	-1 [-13, 10]	7.8
Function f_{3d}		-6 (9)	-9 [-21, 5]	10.9
Function f_{4d}	T4 (46.7±1.7)	0 (5)	1 [-7, 9]	4.9
Function f_{3d}		-9 (6)	-8 [-19, -1]	10.7

RD … Relative deviation; RMSE … root mean square error; TIA … Time-integrated activity; MS … Model selection; NLME … Non-linear mixed-effects modelling; STP … single-time-point
